# Predictors of patient length of stay post stroke rehabilitation

**DOI:** 10.4314/ahs.v23i2.63

**Published:** 2023-06

**Authors:** Thea Bijl, Witness Mudzi, Nicolette Comley-White

**Affiliations:** Department of Physiotherapy, Faculty of Health Sciences, University of the Witwatersrand

**Keywords:** Length of stay, predictors, rehabilitation, stroke

## Abstract

**Background:**

There is little research on length of hospital stay (LOS) in patients post stroke in South African rehabilitation facilities. As LOS is an important indicator of cost-of-care, this information may be useful to all stakeholders.

**Objectives:**

To determine the predictors of hospital LOS in patients post stroke rehabilitation.

**Methods:**

A retrospective file review of 243 patients.

**Results:**

Patient functional ability was measured using the Functional Independence Measure (FIM). Predictors of LOS were determined with multiple regression analysis. The median admission and discharge FIM scores were 43 (range: 16-119) and 75 (range: 16-120) points respectively. The median LOS was 43 (range: 3-112) days. Predictors of LOS were premorbid psychiatric conditions, impaired speech, requiring oxygen support, the development of pneumonia and admission FIM motor score, with admission FIM motor score being the strongest individual predictor of LOS (41%).

**Conclusion:**

Admission FIM score had an influence on patient outcomes and LOS. Patients with higher admission FIM motor scores may be able to participate in rehabilitation better and thus have shorter LOS. Being able to predict LOS on admission allows facility administrators to manage bed occupancy, human and clinical resources in post stroke rehabilitation.

## Introduction

Stroke is the main cause of disability in South Africa^1^ and post stroke patients require rehabilitation to reduce disability and promote functional recovery^2^, ^3^. Inpatient rehabilitation facilities provide patient rehabilitation over a period of days or weeks. In South Africa, patients with stroke can access healthcare through government facilities or private facilities, the latter of which is self-funded either independently or through a medical insurance. Thus, financial resources influence a patient's access to private healthcare and the length of stay (LOS) can be affected by this. Another factor than can affect LOS is the patients' functional outcomes on admission. Patient functional outcomes may be measured using standardised outcome measures such as the Functional Independence Measure (FIM)^4^.

Little is known about LOS and the predictors in the South African stroke population, especially within the private health sector, hence there is a need for more comprehensive observational and descriptive studies in a South African rehabilitation context. This can be used in future to recommend LOS and motivate to all funders involved an estimated time. The disparities between the South African rehabilitation service delivery and rehabilitation services abroad necessitates the need for further research in this area. Thus, the aim of this study was to determine the factors that predict hospital LOS in patients admitted to a private, sub-acute rehabilitation setting in Gauteng, South Africa, post stroke.

## Methods

This study was a retrospective file review. Data which included referral reports, standard hospital admission forms, therapists' admission reports, patient progress notes and laboratory results were gathered from files for patients admitted between 1 January 2015 and 31 January 2017.

The files were sampled from a private, sub-acute rehabilitation facility in Gauteng, South Africa. The facility has 102 adult beds and receives patient referrals from acute hospitals all over Gauteng, from both the public and private sector. Patients were admitted to the facility if they were medically stable, weaned from ventilators and able to participate in three hours of therapy per day. Patients were ideally discharged from the facility when they were able to return safely to their homes. Patients' LOS may have been limited by the financial coverage that their medical insurance scheme provided.

The FIM was used to measure functional gains at this facility. The FIM evaluates a patient's ability to perform 18 motor and cognitive tasks which are rated on an ordinal scale, ranging from 18 (lowest) to 126 (highest)^5^. The final score on the FIM indicates a patient's level of independence, with lower score indicating a higher burden of care, and vice versa for a higher score. The FIM data that was gathered pertained to scores on admission (including the total score, motor and cognitive sub-scores), scores on discharge, FIM gain for total and sub-scores of the FIM, speech and language abilities, respiratory function, swallowing abilities and continence on admission.

Participants were included in the study if they were over the age of 18 and were admitted with first-time stroke. Participants were excluded from the study if they died prior to discharge (as this would influence the LOS and the change in FIM analyses), had a previous stroke (due to the potential influence this would have on the patient's level of function prior to discharge), suffered from dementia (due to Alzheimer's disease or other aetiology) or had files with incomplete data (specifically admission and discharge dates and FIM scores). While it would have been of interest to note the number of patients who were excluded and describe their demographics and their possible impact on the findings, the files of these patients were not included in the study at the time of data collection.

Stroke severity was not described in terms of any standardised outcome measure, however functional dependence at admission (as measured with the FIM) determined the level of burden and thus was the best indicator of stroke severity in this study. The reason for discharge was not documented in patient reports or files and could thus not be included in the study despite its importance in determining LOS.

Ethical clearance was granted by the University of the Witwatersrand Human Research Ethics Committee (M160936) and permission was obtained from the research committee of the rehabilitation facility.

### Data analysis

Descriptive statistics (median and range values) were used to describe the sample, their functional outcomes and their LOS as data were not normally distributed.

Preliminary investigations were done to determine if there were differences in patient discharge FIM scores and LOS based on patients' admission FIM scores. The sample was thus divided into three groups based on level of impairment, namely mild, moderate and severe disability as proposed by various authors^6^,^7^,^8^. To determine if there were differences between groups, median values of sample groups were compared using the Kruskal-Wallace One Way ANOVA on rank and Dunn's Method tests.

A multiple regression analysis was done to determine the predictive value of clinical, demographic and functional variables on LOS. To build a model for predicting LOS, demographic, clinical and functional variables that may have had a theoretical link to LOS were identified from the literature and clinical judgement. Bivariate correlations were done with all the independent variables (IVs) to the dependent variable (DV), LOS, in order to determine if there were significant relationships.

To check for multicollinearity, correlations of IVs to IVs were done, as well as variance inflation factor (VIF) and tolerance statistics. If two IVs were highly correlated, the redundant variable was removed. Bivariate correlations were done with each of the demographic, clinical and functional variables with LOS. A stepwise multiple regression model with different combinations of IVs was used to determine if the variables were predictors of hospital LOS. Instead of relying on what is already known in the literature, we decided on looking at all the variables for which we had to see if the same factors would also come out as predictors of hospital length of stay. The selected model had the smallest significant F-value (18.3), indicating that the probability of the null hypothesis (that all coefficients are 0) being true was 18.3%. The selected model showed the best combination of independent variables for predicting LOS in this sample. Once the individual regressions for each IV were done, coefficient values, t-statistics and p-values were obtained, and then an ANOVA was done to get f-values and p-values.

R-squared, r-squared adjusted, r-squared predicted and VIF values were used to choose the best model. A residual analysis was done to identify atypical data points and remove outliers.

## Results

### Description of demographic and clinical characteristics

The study included 243 patient files. Patient median age was 61.0 (range: 20-87) years, with 56% (n=136) of the study sample being female. The majority of the strokes were ischaemic (73.3%; n=179). Table 1 presents further information about the demographic and clinical characteristics of the study sample.

**Table 1:** Demographic and clinical characteristics of the study sample (n= 243)

Demographic variables	Sub-category	Number of patients (%)
Gender	Male	107 (44.0)
	Female	136 (56.0)

Medical insurance	Insured	238 (97.9)
	Uninsured	5 (2.1)

Marital status	Single	41 (16.9)
	Married	151 (62.1)
	Widowed	51 (21.0)

Level of independence prior to stroke	Independent	234 (96.3)
	Dependent	9 (3.7)

Employment status	Employed	130 (53.5)
	Unemployed	7 (2.9)
	Retired	103 (42.4)
	Student	3 (1.2)
Stroke side	Left sided stroke	111 (45.7)
	Right sided stroke	125 (51.4)
	Bilateral stroke	7 (2.9)

Stroke type	Ischaemic	179 (73.7)
	Haemorrhagic	64 (26.3)

Respiratory support required	None	225 (92.6)
	Require oxygen	17 (7.0)
	Tracheostomy	1 (0.4)

Swallowing function	Normal	187 (77.0)
	Unsafe	56 (23.0)

Diet	Normal	143 (58.8)
	Soft	69 (28.4)
	Nasogastric tube	7 (2.9)
	Percutaneous endoscopic	24 (9.9)
	gastrostomy tube	

Speech ability	Normal	103 (42.4)
	Affected	140 (57.6)

Type of speech disorder	Apraxia	49 (35)
	Aphasia	46 (32.9)
	Dysarthria	63 (45)

Presence of unilateral spatial neglect		44 (18.1)
Continence	Continent	119 (49.0)
	Incontinent	124 (51.0)

Complications were documented in the hospital files of 71.2% (n=173) of the patients. The most common complications were pneumonia (20.8%), depression (19.7%) and pressure sores (18.5%). The most common co-morbidities were hypertension (66.3%, n=161), hypercholesterolaemia (35.4%, n=86), cardiac conditions (33.3%, n=81) and diabetes mellitus (27.2%, n=66). Patients could present with more than one complication or co-morbidity. Table 2 presents further information on the comorbidities.

**Table 2:** Co-morbid conditions amongst the study sample (n= 243)

Co-morbid condition	Number of patients (%)
Hypertension	161 (66.3)
Hypercholesterolaemia	86 (35.4)
Cardiac condition	81 (33.3)
Diabetes	66 (27.2)
Arthritis	49 (20.2)
Auditory impairment	47 (19.3)
Respiratory condition	44 (18.1)
Visual impairment	37 (15.2)
Orthopaedic condition	30 (12.2)
Epilepsy	25 (10.3)
Human immunodeficiency virus	23 (9.5)
Renal condition	23 (9.5)
Hysterectomy	23 (9.5)
Gastric condition	17 (7.0)
Psychiatric condition	15 (6.2)
Thyroid condition	15 (6.2)
Prostate condition	12 (4.9)
Neoplastic condition	6 (2.5)
Substance abuse related conditions	5 (2.1)

### Description of functional outcomes of the study sample (FIM data)

A secondary aim of the study was to determine patients' functional outcomes using the FIM. The median admission FIM score was 43 points, the median discharge score was 75 points and the median FIM change score was 21 points (Table 3). To determine if admission FIM score influenced functional outcome, the sample was divided into three groups, those with mild, moderate and severe disability. For each FIM admission group, the medians and ranges were calculated for each of the following two variables: discharge FIM score and FIM change score. Patients in the low FIM admission score group (<40) had low median discharge FIM scores (46), and patients in the high FIM admission score group (>80) had high median discharge FIM scores (100). The FIM change appeared to be greatest in the patients with moderate impairments (25 points), while mildly impaired and severely impaired patients had smaller median FIM change scores (Table 4). The biggest contributor to the FIM scores was the motor component for all the three categories.

**Table 3:** FIM scores of the study sample (n= 243)

FIM scores	Sub-categories	Median	Range
Total FIM score	Admission FIM	43.0	16–119
	Discharge FIM	75.0	16–120
	FIM change	21.0	-23–82
Motor FIM score	Admission FIM	26.0	2–89
	Discharge FIM	52.0	11–90
	FIM change	21.0	-23–82
Cognitive FIM score	Admission FIM	15.0	5–35
	Discharge FIM	22.0	5–54
	FIM change	4.0	-13–33

**Table 4:** Median discharge FIM scores and FIM change scores of the admission FIM groups of the study sample (n= 243)

Admission FIM group	Median Discharge FIM score	Range	Median FIM change	Range
<40 (severe impairment) n=109	46.0	16.0 – 100.0	20.0	-14.0–82.0
40-80 (moderate impairment) n=108	83.0	27.0 – 120.0	25.0	-23.0–64.0
>80 (mild impairment) n=28	100.0	92.0 – 119.0	11.0	0.0–29.0

For every FIM admission group, the Dunn's Pairwise Multiple Comparison Method showed that the FIM discharge scores were statistically significantly different (p< 0.05), and the same was true for all pairwise comparisons of FIM change scores, except in the <40 and 40-80 group (p> 0.05). However, at the group level tested in this study, the results were not conclusive, as the ranges in score were very large in each group, and further analysis was beyond the scope of this study.

### Description and prediction of LOS

The LOS of patients ranged between three and 112 (median=43) days. Table 5 presents the variables that significantly correlated with LOS. The four variables that had the strongest correlation with LOS were the development of pneumonia (r= -0.22), admission FIM cognitive score (r= -0.29), the admission FIM motor score (r= -0.43), and the admission FIM total score (r= -0.42). The variables incorporated into the most predictive model were: admission FIM motor score, the development of pneumonia, requiring oxygen support, impaired speech on admission, and the presence of a premorbid psychiatric condition (Table 6). The variance in the LOS explained by the model was 26.3%.

**Table 5:** Bivariate analysis of IVs that were correlated with LOS at p<0.1

Independent Variable (IV)	N	R-value	Shared variance (r2) (%)	p-value (p< 0.1)
Presence of cardiac condition	81	-0.16	2.6	0.010
Valve replacement	16	0.11	1.2	0.083
Presence of orthopaedic condition	30	-0.14	2.0	0.032
Presence of a psychiatric condition	15	-0.14	2.0	0.030
Requiring oxygen support	17	0.12	1.4	0.061
Affected speech at admission	140	0.19	3.6	0.003
Apraxia at admission	49	0.12	1.4	0.062
Incontinence at admission	124	0.12	1.4	0.059
Development of pneumonia	36	-0.22	4.8	<0.001
Admission FIM motor score	243	-0.43	18.5	<0.001
Admission FIM cognitive score	243	-0.29	8.4	<0.001
Admission FIM total score	243	-0.42	17.6	<0.001

**Table 6:** Multivariate analysis coefficients for the selected model

Factor	Coefficient			t	Significance (p-value)	95% Confidence Interval for B	

	Unstandardised Coefficients		Standardised Coefficients			Lower Bound	Upper Bound

	B	Std. Error	Beta (b)				
(Constant)	53.17	2.28	-	23.36	-	48.7	57.7
Admission	-0.38	0.05	-0.41	-7.31	<0.01	-0.48	-0.28
FIM motor score							
Pneumonia	-9.14	2.68	-0.19	-3.42	0.001	-14.41	-3.87
Oxygen support	9.64	3.71	0.15	2.60	0.010	2.32	16.95
Impaired speech on admission	5.14	1.90	0.15	2.70	0.007	1.40	8.90
Psychiatric conditions	-8.33	3.92	-0.12	-2.13	0.035	-16.10	-0.61

The IV that was the largest individual contributor to predicting LOS was the admission FIM motor score when all other values were controlled for (b= -0.41 i.e., 41%). This was followed by the development of pneumonia (19%), the requirement of oxygen support (15%), having a speech impairment at admission (15%) and the presence of a premorbid psychiatric condition (12%). Post the bivariate analysis, all the IVs' whose correlations with LOS were at p< 0.1 were included in the multiple regression analysis. A one-point increase in admission FIM motor score reduced LOS by almost a third of a day (B= -0.38). The development of pneumonia as a medical complication reduced LOS by just over nine days (B= -9.14). Requiring additional oxygen support (nasal cannula or facemask) increased LOS by almost 10 days (B= 9.6). Having impaired speech on admission (apraxia, dysarthria, aphasia or a combination) increased LOS by approximately five days (B= 5.14), and having a premorbid psychiatric condition reduced LOS by more than eight days (B= -8.33) (Table 6).

## Discussion

This study aimed to determine the predictors of hospital LOS in patients admitted to a private, sub-acute rehabilitation facility in South Africa, post stroke. The median age of patients in this study (61.0 years, range 20-87 years) was slightly younger than the ages reported for patients admitted to rehabilitation centres in Italy, the United States and Canada (68-73 years)^9^-^12^. Wang et al. (2016)^13^ reported an increased risk of stroke in younger adults in middle and low-income countries. This could explain why this study's patients were slightly younger than those recorded by studies in developed countries. In an acute, state-run tertiary hospital in South Africa, the mean age of patients studied post stroke was 59.8 years^14^, and in a specialised state-run rehabilitation facility the mean age of stroke patients was 53 years^15^. The similarity in other South African facilities supports the theory that the high number of stroke risk factors may be responsible for the younger age at which patients have strokes in developing countries^16^-^20^.

The median LOS for patients at this facility was 43 days. It is also similar to the LOS reported by two studies of patients post stroke at a specialised state-run rehabilitation facility in the Western Cape (South Africa) with 62 days and 51.6 days respectively^15^-^16^.

Most patients had admission FIM scores between 40-80 points and could be classified as moderately to severely impaired^6^, ^17^. The median admission FIM score of 43 is quite low when compared to other samples of post stroke patients selected for similar studies, such as Black-Schaffer and Winston (2004)^12^ and Valach, Selz and Signer (2004)^18^ with mean admission FIM scores of 60.7 and 79.4 respectively. These lower admission scores are similar to those recorded in patients receiving rehabilitation post stroke across 23 private hospitals in South Africa (between 40-65 points)^19^. The admission criteria may be similar for private, South African rehabilitation facilities resulting in similar mean admission scores for patients post stroke. The reason for differing scores abroad could stem from the facility policies, i.e., more stringent admission criteria may limit more severely impaired patients from accessing rehabilitation as they are seen as unlikely to make sufficient functional gains. They are instead referred to nursing facilities^20^-^21^. Additionally, the prescribed minimum benefits stipulated in South African medical insurances covers stroke, thus influencing the access to private healthcare facilities for patients regardless of FIM scores.

It is a common finding that admission FIM score is the strongest predictor of discharge FIM score^9^, ^11^-^12^, ^22^-^24^. The results of this study support these findings. For every FIM admission group in this study, pairwise comparisons showed that the FIM discharge scores were statistically significantly different (p< 0.05). Patients who come in with low admission FIM scores had significantly lower discharge FIM scores when compared to those who had moderate or high FIM scores on admission. Patients had markedly different outcomes based on their admission scores, and thus the admission FIM score of a patient may be useful in predicting their functional outcomes (however, other potential influencing factors must still be taken into consideration, for example, premorbid function).

The change in FIM scores appears to be greatest in the patients with moderate impairments, while mildly impaired and severely impaired patients have smaller changes in median FIM scores. Very severely impaired patients may not have as much potential for improvement as those with moderate impairment, and those with the highest FIM scores may not show the same gains due to the ceiling effect of the FIM in highly functional patients^9^.

Admission functional score (measured with the FIM or Barthel Index (BI)) have been found to be a predictor of LOS in several studies^10^, ^25^-^29^. Patients with more severe functional impairments require longer LOS to be ready for home discharge. The FIM motor score is the most suitable sub-scale for evaluating patients' mobility outcomes at discharge and monitoring a change in mobility from admission to discharge^30^. In this study, an increase in admission FIM motor score was found to reduce LOS (a one-point increase reduces LOS by 0.4 days), in line with other studies^29^, ^31^. It stands to reason that patients who have greater mobility are more able to participate in rehabilitation transfers and activities of daily living (ADLs). They make more rapid gains with therapy and may be ready for discharge earlier^25^. Patients who cannot mobilise need longer LOS to promote maximal functional improvement^32^.

The presence of a pre-morbid psychiatric condition decreased LOS by just more than eight days. This was a novel finding. It may be that patients with psychiatric conditions are less compliant with rehabilitation and may not make sufficient gains with therapy to justify a prolonged stay in a rehabilitation facility. They may thus be discharged sooner to institutional care^33^. These patients may also already have care structures in place at home and do not have to wait for placement^33^.

In this study sample, impaired speech was a common problem, affecting 57.6% of patients. Aphasia increases rehabilitation LOS in patients post stroke^31^, and this study found a similar trend. In the regression analysis, the presence of any speech impairment on admission was an independent predictor of LOS when other variables were controlled for. Impaired speech on admission prolonged LOS by approximately five days. Patients with impaired communication abilities may have more difficulty participating in rehabilitation and making their needs known^34^. They may also struggle to follow instructions which may hinder learning of ADLs^35^. They may need a longer LOS, to achieve the same milestones as patients without speech impairments^31^.

The development of pneumonia was found to reduce LOS by just over nine days in this sample. Other studies have found that there is a negative correlation between pneumonia and LOS, likely because pneumonia is a common cause of mortality^36^-^37^. It could be that patients in a rehabilitation setting who make poor functional gains due to illness and the inability to participate in rehabilitation, are discharged sooner to institutional care or back to acute facilities, resulting in a shorter LOS in the rehabilitation setting. It was beyond the scope of this study to establish this but may be of interest for future studies.

### Implications and recommendations

The model as a whole, as well as the individual predictors, may be useful in identifying the “high risk” characteristics leading to reduced or prolonged LOS in patients post stroke. This information is vital as it aids clinicians in developing the best strategies for the prevention of complications. It may also guide the development/adaptation of admission criteria of the rehabilitation facility. Patients with premorbid psychiatric conditions may be more appropriately managed with out-patient or home-based care, or referral to an appropriate nursing facility as they may not make sufficient gains to warrant prolonged in-patient rehabilitation. Patients who still require oxygen therapy may be better managed with longer stays in acute facilities to ensure that their cardiorespiratory function and endurance is greater prior to referral. This may improve their potential for participation in rehabilitation.

Being able to predict LOS at the earliest stage possible (i.e., on admission) may also be useful for facilities who need to manage bed occupancy and resources. Low admission FIM motor scores on admission may be used to motivate for additional funding and longer LOS from medical insurance schemes. The longer stays may give patients additional time to improve their functional outcomes and prospects for home discharge with fewer impairments.

As a retrospective review, this study was based on the quantity and quality of information available in patient files. Much of the medical and clinical data were reported subjectively by patients or their family members to nursing staff on admission to the facility. This could have influenced the reliability of this study's findings. Additionally, the stroke severity was not recorded for this study and it would have been of value to investigate the relationship between the stroke severity and functional dependence. A further limitation of the study is that it was based at a single site and is thus not generalizable to the whole population. These limitations can be used to inform future studies in this field, and a further recommendation would be to investigate this study's outcomes separately for patients who improve functionally and those who decline over time, to establish if the profile of predictors would be different.

## Conclusion

The predictors of LOS identified in this study included admission FIM score, the development of pneumonia, requiring oxygen support, having a speech impairment at admission and the presence of a premorbid psychiatric condition. The overall variance in LOS predicted by the multiple regression model using this combination of variables was 26.3%.

The IV that was the largest individual contributor to predicting LOS was admission FIM motor score. Patients who had greater mobility were more able to participate in rehabilitation and ADLs. They made more rapid gains with therapy and were discharged earlier. A one-point increase in admission FIM motor score reduced LOS by almost a third of a day. These predictors need to be taken into consideration by facility administrators when managing bed occupancy and human and clinical resources in post stroke rehabilitation.
